# Supplementary material for: Cis-regulatory evolution of the recently expanded Ly49 gene family
Source: Nat Commun. 2024 Jun 6;15:4839. doi: 10.1038/s41467-024-48990-y (PMC11156856; doi:10.1038/s41467-024-48990-y)
Supplement: Supplementary file 1 — Supplementary Information [file 41467_2024_48990_MOESM1_ESM.pdf]

Cis-regulatory evolution of the recently expanded *Ly49* gene family

Supplementary Figures

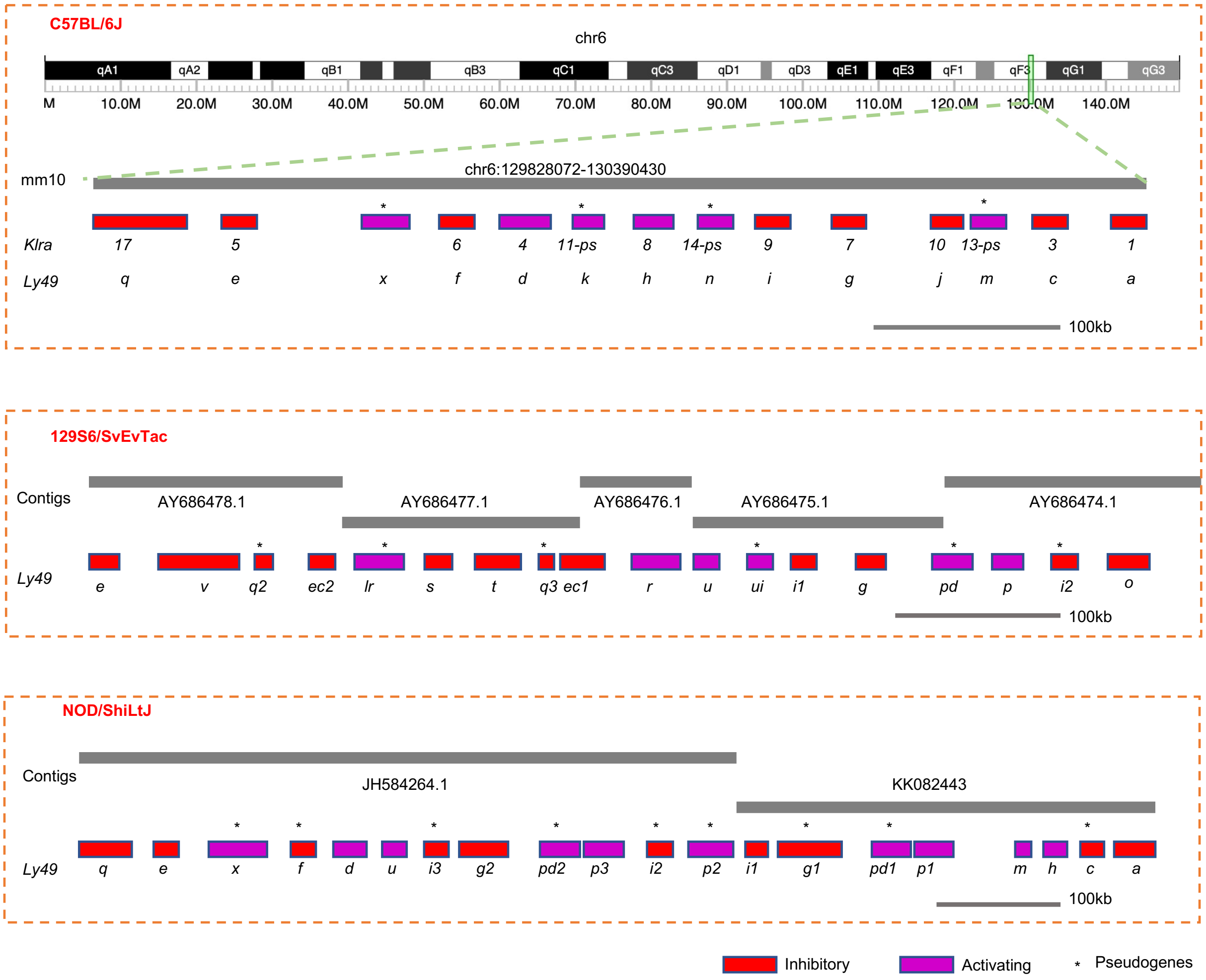

Supplementary Fig. 1 | The *Ly49* clusters in B6, 129 and NOD mice. Top: Location of the *Ly49* locus on mouse chromosome 6 (mm10). *Ly49* genes from B6, 129 and NOD strains were assigned as activating or inhibitory according to previous publications[1–3]. Pseudogenes are labeled with *. For the B6 strain, the corresponding *Klra*-based naming is also provided where available.

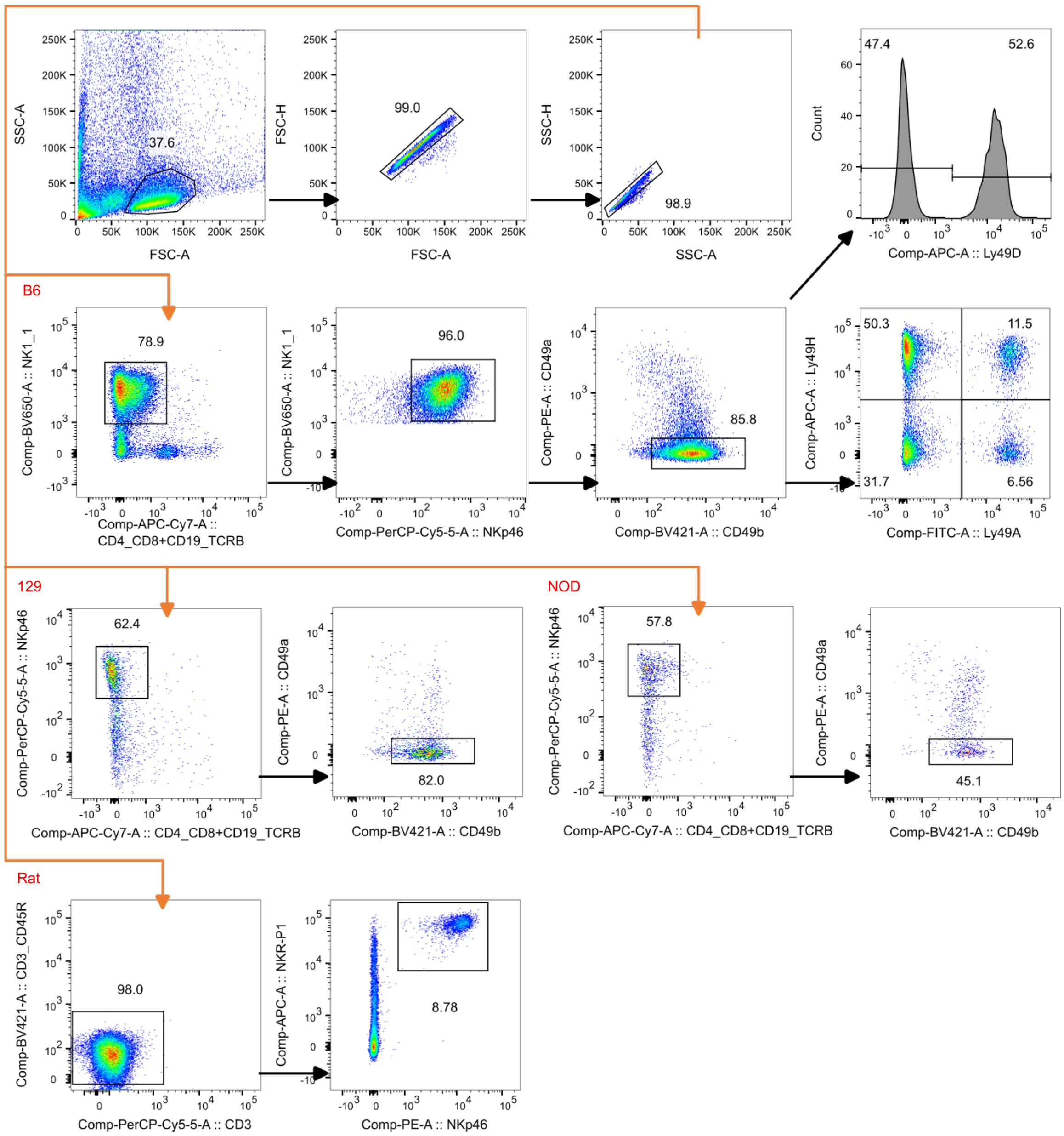

Supplementary Fig. 2 | Representative NK gating strategies used in sorting. Numbers indicate the percentages of cells within indicated gates. Knockout mouse models generated in this study and CB6F1/J samples were also sorted using the B6 gating strategy.

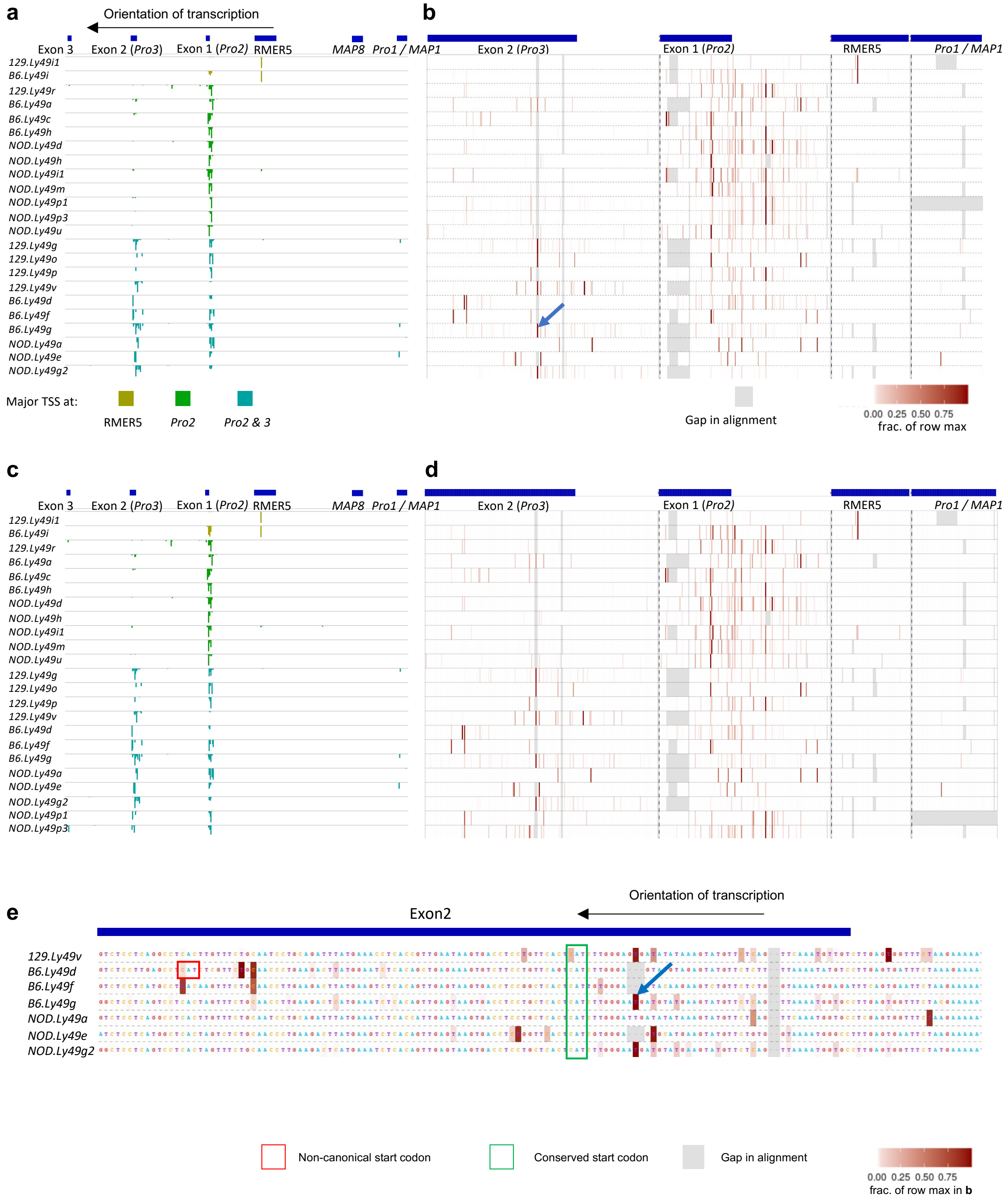

Supplementary Fig. 3 | Shared and diverse *Ly49* transcription start site (TSS) profiles. B6, 129 and NOD indicate the 3 mouse strains.

a, Distribution of *Ly49* TSS signals along the *Ly49* consensus sequence (see Methods). No MAPQ filter was applied.

b, Zoomed-in single base pair resolution view of nanoCAGE signals at *Pro1*, *Pro2*, *Pro3*, and RMER5 elements. Each column represents one nucleotide. Color intensity represents nanoCAGE signal intensity. No MAPQ filter was applied. Blue arrow indicates the *Pro3* TSS conserved among *Ly49a/g/o* genes, but not other *Ly49* genes.

c-d, Same as **a-b**, except that only reads passing MAPQ = 255 were used.

e, A further zoomed-in view of exon 2 (*Pro3*) TSS profiles from **b**. *Ly49* genes are on the negative strand. As a result, start codons are CAT. Blue arrow indicates the *Pro3* TSS conserved among *Ly49a/g/o* genes, but not other *Ly49* genes. Source data are provided as a Source Data file.

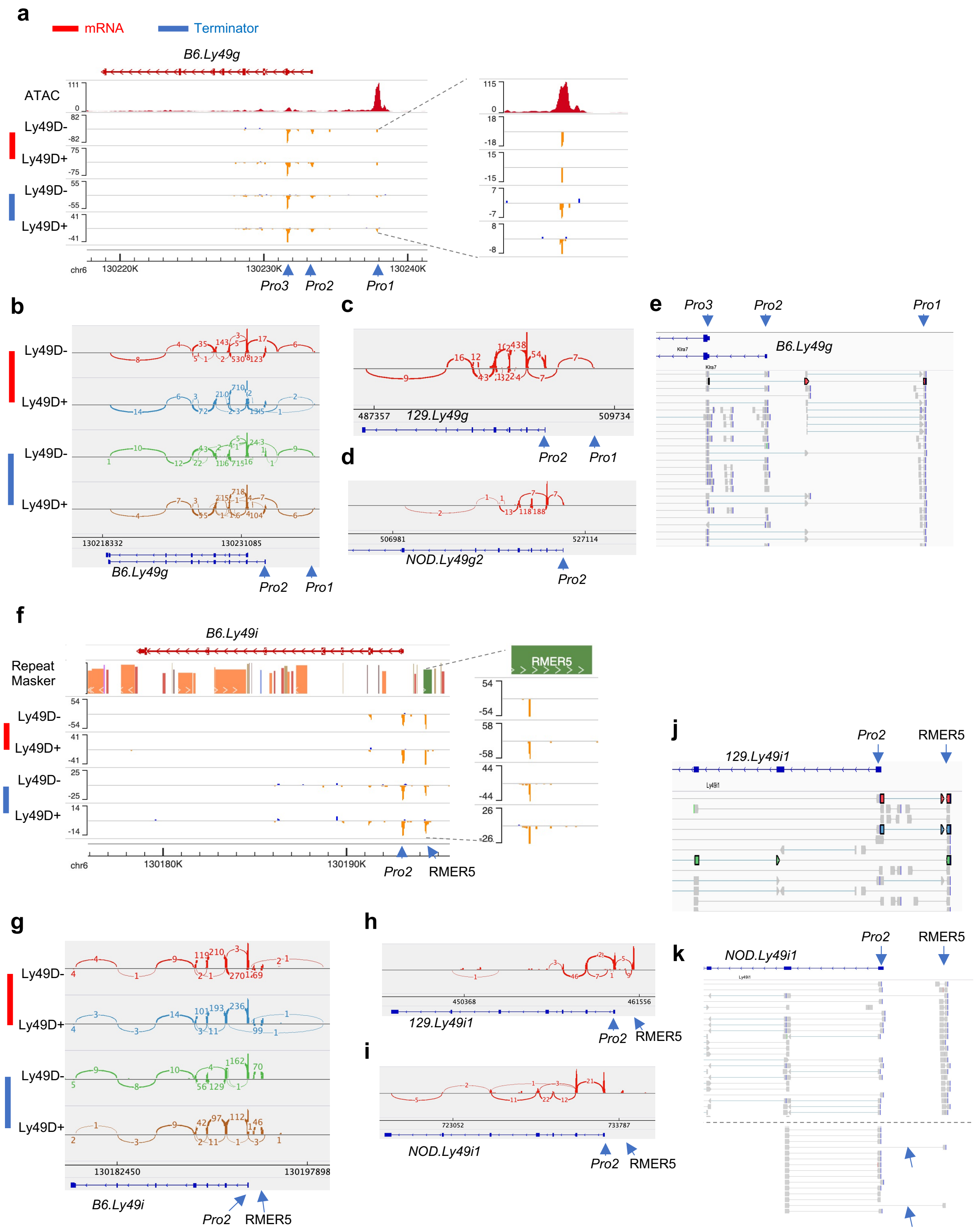

Supplementary Fig. 4 | Other noncanonical TSSs of *Ly49* genes. Based on reads passing the filter of MAPQ = 255. B6 NK cells were sorted into Ly49D^{+} vs Ly49D^{-} subsets. mRNA and Terminator represent 2 similar nanoCAGE protocols (Methods).

a, nanoCAGE TSS signal profiles of *B6.Ly49g*.

b-d, Sashimi plots of *Ly49g* genes.

e, Raw reads view of nanoCAGE data at *B6.Ly49g*. One of the reads initiating from *Pro1* and splicing into gene body was highlighted in red.

f, nanoCAGE TSS signal profiles of *B6.Ly49i*.

g-i, Sashimi plots of *Ly49i* genes.

j, Raw reads view of nanoCAGE data at *129.Ly49i1*. Two of the reads initiating from RMER5 and splicing into exon 1 were highlighted in red and blue, respectively. One read pair with one mate overlapping RMER5 and the other mate spanning exons 2 and 3 was highlighted in green.

k, Raw reads view of nanoCAGE data at *NOD.Ly49i1*. Two read pairs with one mate overlapping RMER5 and the other mate overlapping *NOD.Ly49i1* exons were highlighted by arrows. Source data are provided as a Source Data file.

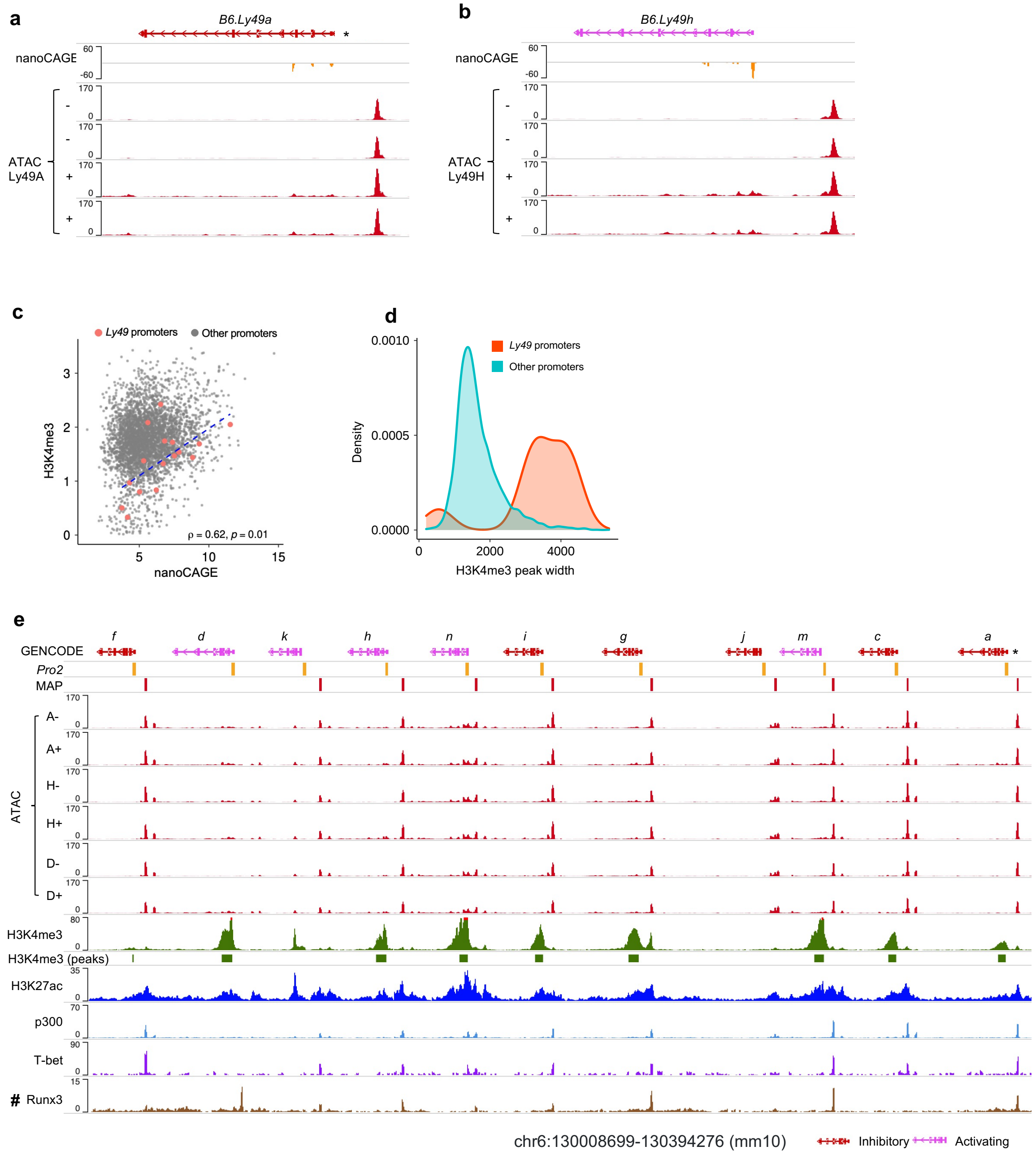

Supplementary Fig. 5 | Mapping *Ly49* promoters and enhancers. * indicates manual curation of the GENCODE (M19) *B6.Ly49a* annotation. The original GENCODE annotation designated *Pro1* as a *B6.Ly49a* promoter, which is not supported by our nanoCAGE data.

a-b, ATAC-seq signal profiles of Ly49A$^+$ vs Ly49A$^-$ NK cells and Ly49H$^+$ vs Ly49H$^-$ NK cells.

c, Scatter plot of H3K4me3 signals vs nanoCAGE signals for each active promoter (Methods). H3K4me3 is the average of 2 samples (GSM4314396[4] and GSM4314407[4]). nanoCAGE is the average of all 4 B6 samples (this study). Pearson correlation was calculated based on *Ly49* promoters only (n = 16 *Ly49* promoters). Both axes were normalized and log2 transformed (Methods). *P* value was calculated from two-tailed Student's *t* statistic.

d, H3K4me3 ChIP-seq peak width distribution at *Ly49* genes (shown in **e**) vs non-*Ly49* genes.

e, Same as **Fig. 1a**, but presenting a zoomed-out view of all B6 *Ly49* genes expressed in conventional NK cells. #: data generated from the ICR mice, but aligned to the B6 genome, due to the lack of an ICR reference genome.

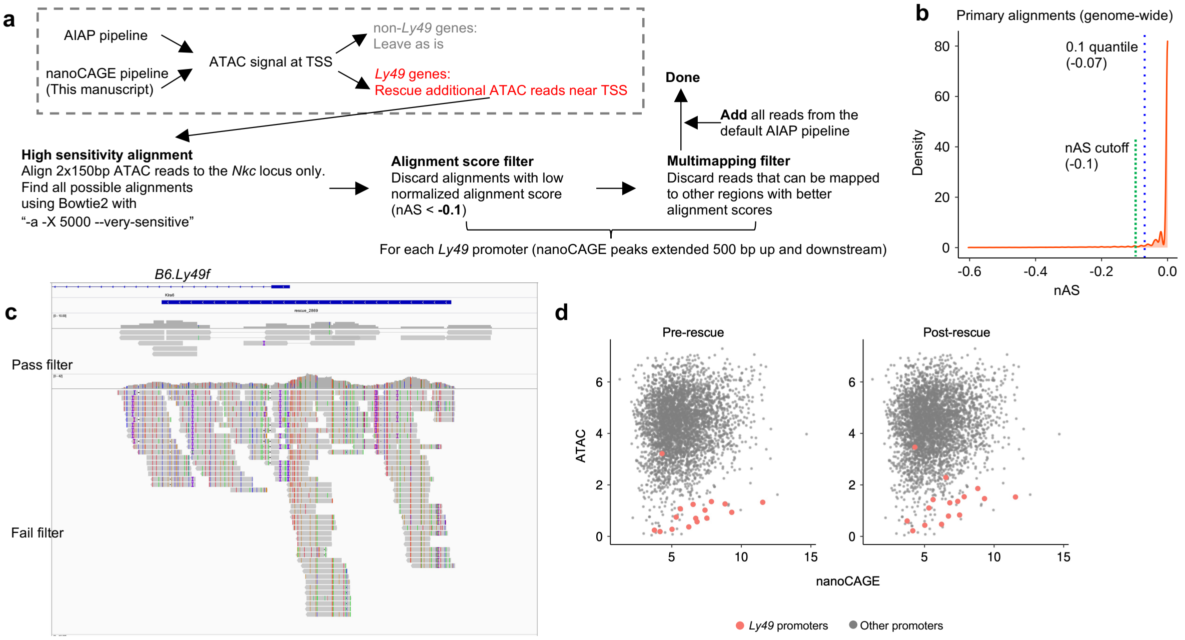

Supplementary Fig. 6 | Mappability cannot fully explain the low ATAC-seq signal at *Ly49* promoters.

a, A schematic representation of the read rescue pipeline (Methods).

b, The distribution of normalized alignment scores (nAS. Methods) calculated from primary alignments across the genome (surrogate for correct alignments). The blue dotted line indicates the 0.1 quantile while the green dotted line indicates the nAS cutoff adopted in this study.

c, IGV view of alignments at the *B6.Ly49f* promoter after high sensitivity alignment. Subsequently, the 2 filters from **a** were applied. Reads that failed filtering exhibited extensive mismatches that piled up at certain nucleotides, suggesting they originated from other *Ly49* promoters.

d, Scatter plots similar to Fig. 1b, contrasting pre and post read rescue.

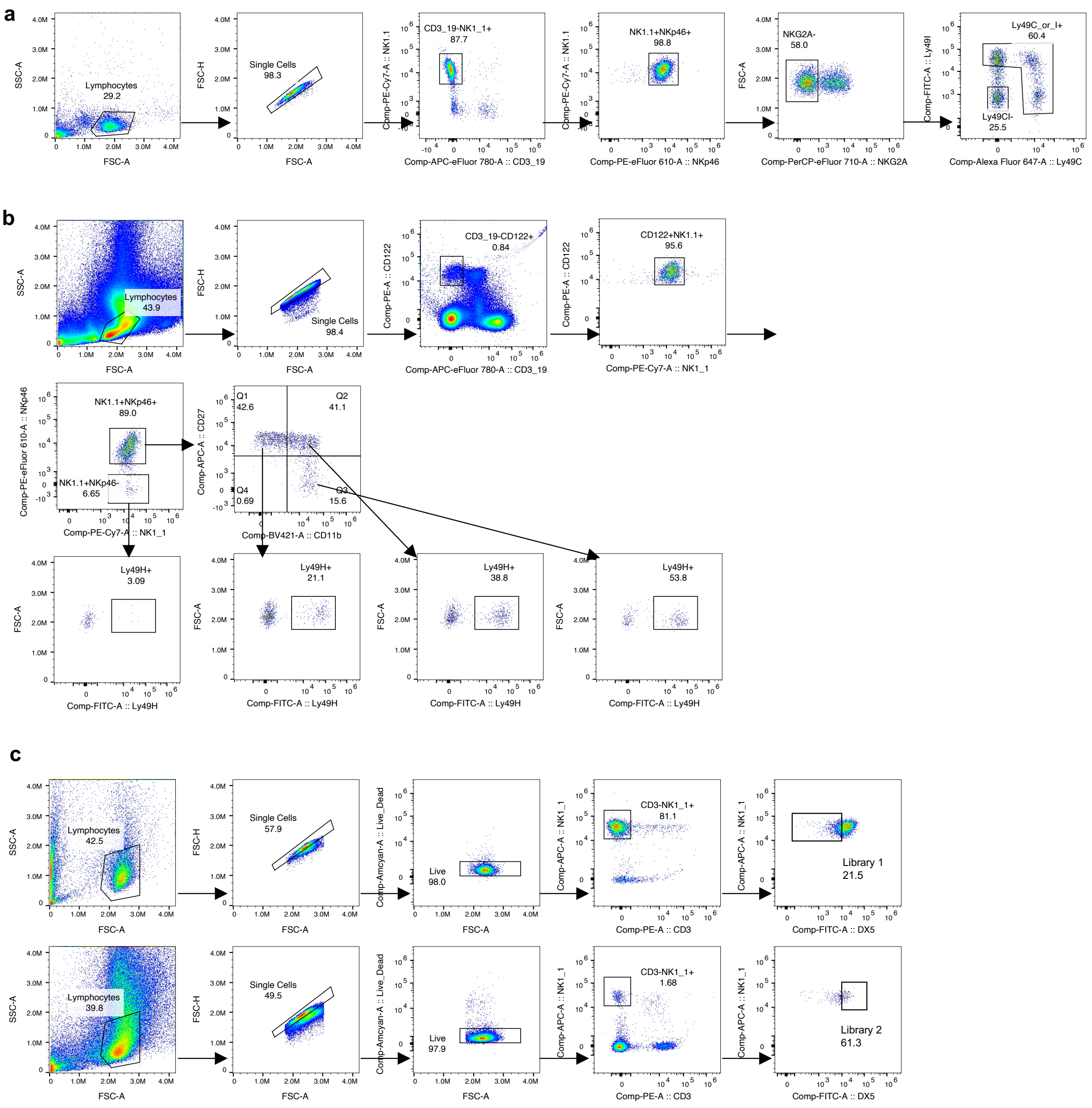

Supplementary Fig. 7 | Gating strategies for bone marrow NK cells and licensed vs unlicensed NK cells

a, Gating strategy for sorting licensed (NKG2A$^-$, Ly49C$^+$ or I$^+$) vs unlicensed (NKG2A$^-$, Ly49C$^-$ and I$^-$) splenic NK cells.

b, Gating strategy for immature NK cells, used to study the effect of *MAP8* KO on *B6.Ly49h* expression in immature NK cells.

c, Gating strategy for sorting bone marrow NK cells for nanoCAGE. Note that library 1 and library 2 are from 2 different animals. For library 1, NK cells were enriched using negative depletion (see Methods) before sorting. For library 2, NK cells were sorted without enrichment.

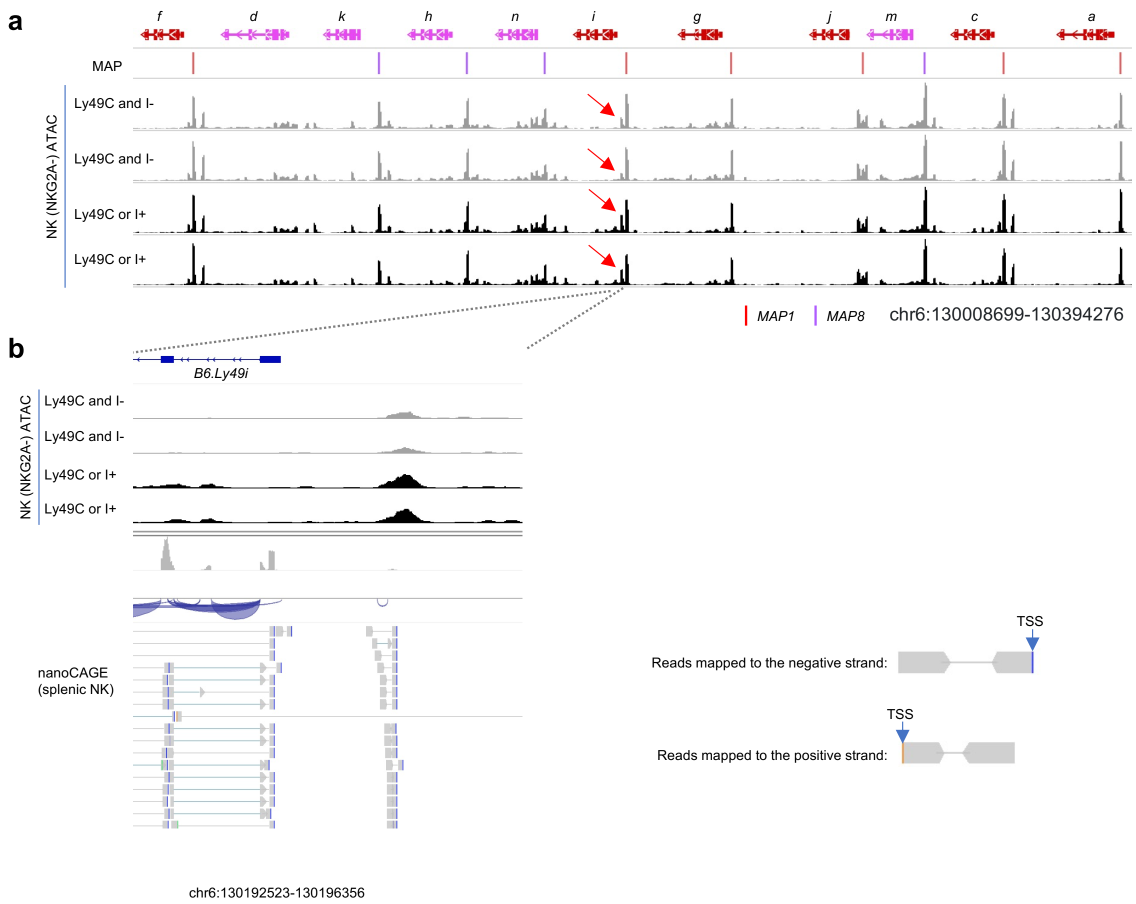

Supplementary Fig. 8 | *Ly49* chromatin accessibility profiles in licensed vs unlicensed NK cells.

a, ATAC-seq profiles of the *Ly49* locus in unlicensed (NKG2A$^-$; Ly49C$^-$ and I$^-$) and licensed (NKG2A$^-$; Ly49C$^+$ or I$^+$) NK cells sorted from B6 spleens. 2 biological replicates for each sample type. Red arrow: an ATAC peak proximal to *B6.Ly49i* more accessible in licensed NK cells.

b, nanoCAGE data showing promoter activity at the aforementioned peak with increased chromatin accessibility in licensed NK cells, suggesting that the increase in chromatin accessibility is likely explained by higher transcriptional activities at the *B6.Ly49i* locus in Ly49I$^+$ cells. Source data are provided as a Source Data file.

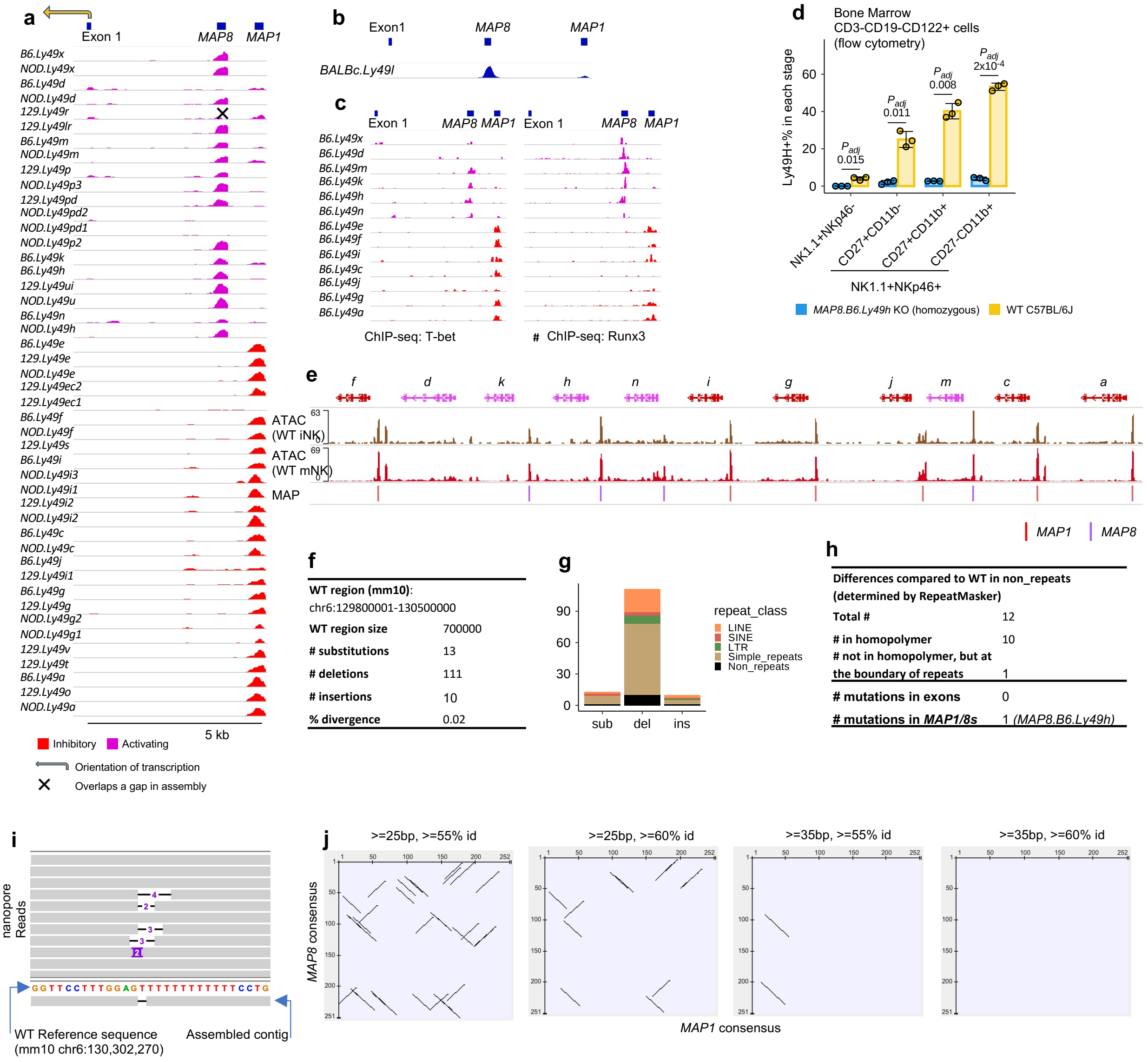

Supplementary Fig. 9 | Inhibitory and activating *Ly49* genes are regulated by two separate sets of proximal CREs.

a, Pileup view of ATAC signals on the coordinates of the *Ly49* consensus sequence. Same as **Fig. 2a**, except that only reads with MAPQ >= 8 were included.

b, NK cell ATAC-seq signals upstream of *BALBc.Ly49l*, the only activating *Ly49* gene encoded in the BALB/c *Ly49* locus (Methods). Data source: GSM5492291[5].

c, Pileup view of ChIP-seq signals. Reads with MAPQ > 8 were included. Data sources: T-bet: GSM4314405[4]; Runx3: GSM1214531[6]. #: Runx3 data was generated from ICR mice, the *Ly49* locus of which could differ from the mm10 reference. Runx3 data was aligned to mm10 due to the lack of reference genome for ICR mice.

d, Bone marrow *B6.Ly49h* expression (percentages of Ly49H$^+$ cells out of all NK cells in each developmental stage) assayed by flow cytometry. n = 3 KO (2 female 1 male) and 3 WT (female) mice. Means (bars) and individual values (points) are shown. Error bars: mean ± s.d. Two-tailed unpaired Student's *t*-test. *P* values are FDR-adjusted.

e, ATAC-seq profiles of bone marrow immature NK cells (iNK) vs splenic mature NK cells (mNK), normalized by sequencing depth. Data source: iNK: GSM2056300[7]; mNK: the B6_Ly49Dp_ATAC_rep1 (Supplementary Data) sample from this study.

f, Differences between the assembled contig versus the WT *Ly49* locus (mm10). % divergence was calculated as the sum of the numbers of substitutions, deletions and insertions, divided by the size of the region on WT coordinates (700 Kb).

g, The genomic locations of the differences from **f** were intersected with the repetitive elements of the genome (determined by RepeatMasker, Methods).

h, Manual curation of differences located in regions not annotated as repeats by RepeatMasker (Non_repeats in **g**).

i, Example of the differences that occurred at DNA homopolymers.

j, Dot plots between *MAP1* and *MAP8* consensus sequences. The default algorithm in UGENE was used to generate the plots based on the indicated criteria. Consensus sequences were generated using all sequences presented in Supplementary Fig. 10-11 according to Methods. Source data are provided as a Source Data file.

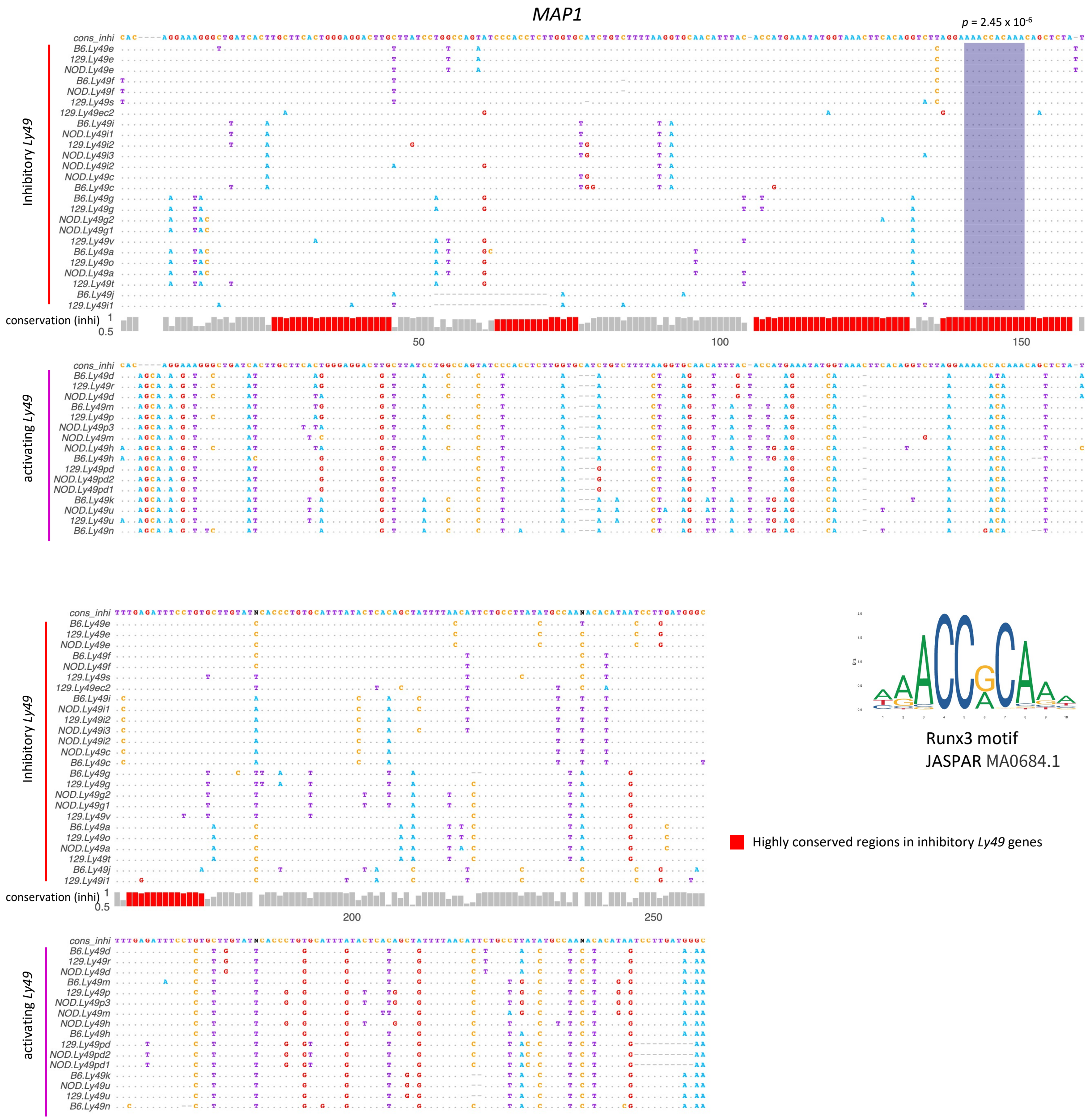

Supplementary Fig. 10 | Alignment of *MAP1* elements from mouse inhibitory and activating *Ly49* genes. Sequences were ordered based on mafft output, which was based on the guide tree of the multiple sequence alignment. *Cons_inhi* indicates the consensus sequence calculated based on inhibitory *Ly49* genes. Conservation was calculated only for sequences from inhibitory *Ly49* genes. Highlighted in red are highly conserved regions defined as sequence segments >= 10bp with >= 90% of the inhibitory *Ly49* paralogs having identical nucleotides at each position. These regions were scanned for the Runx3 motif (Methods). *P*-value (unadjusted) was calculated from alignment scores using dynamic programing as a part of FIMO[8].

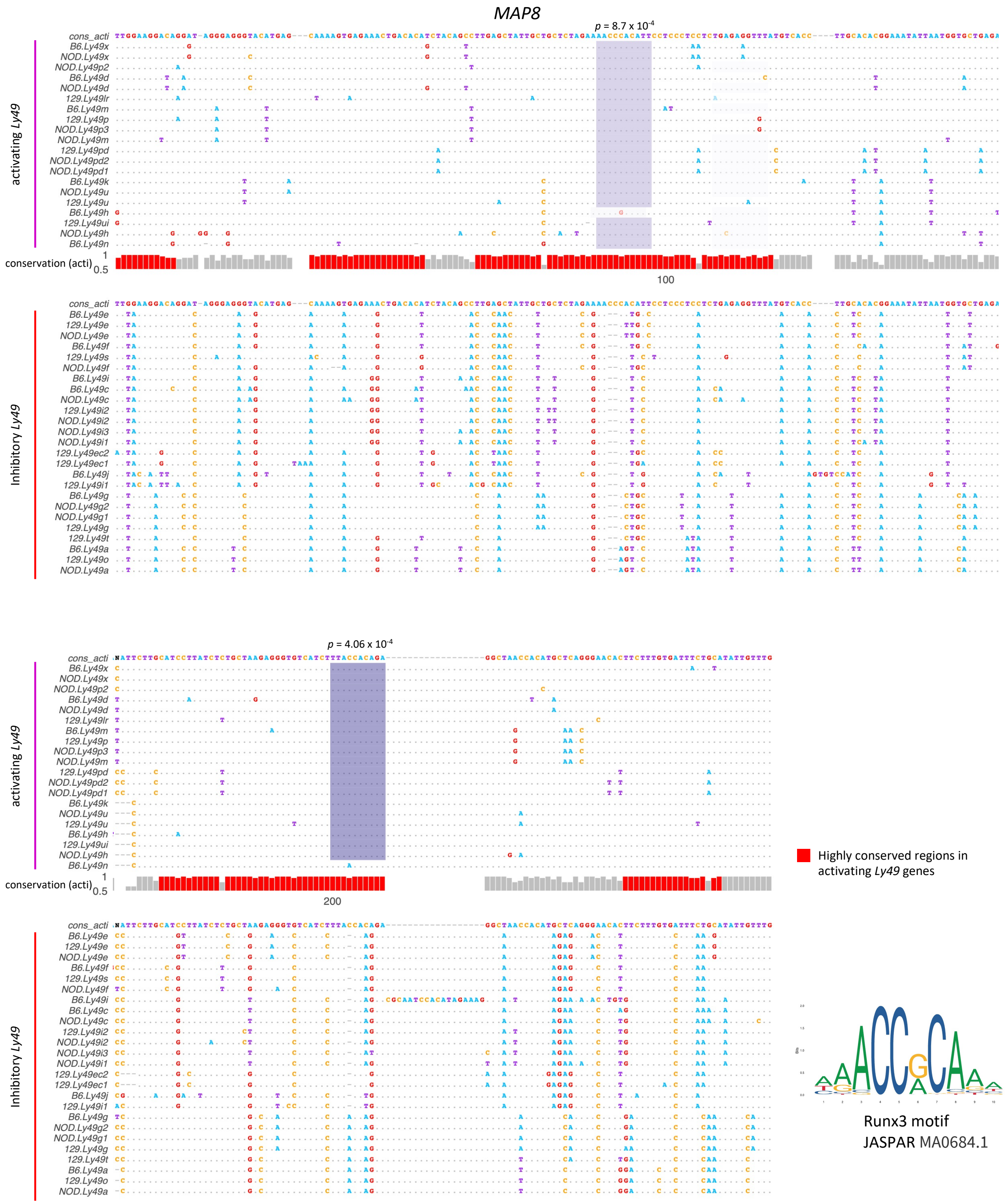

Supplementary Fig. 11 | Alignment of *MAP8* elements from mouse activating and inhibitory *Ly49* genes. Same as Supplementary Fig. 10, but consensus sequence and conservation were calculated based on activating *Ly49* genes. *P*-value (unadjusted) was calculated from alignment scores using dynamic programing as a part of FIMO[8].

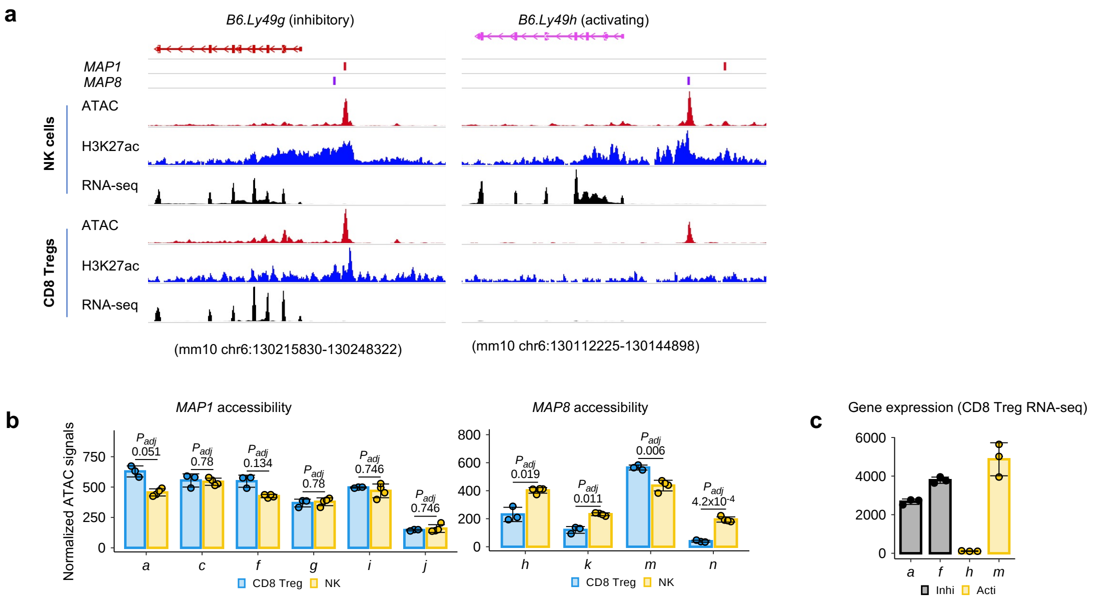

Supplementary Fig. 12 | *MAP1/8* chromatin accessibility in CD8 Tregs.

a. ATAC, H3K27ac, and RNA-seq profiles of the inhibitory *B6.Ly49g* and activating *B6.Ly49h* in NK cells vs CD8 Tregs. Each track is normalized to the same max in view. Data source: NK ATAC: B6_Ly49Dp_ATAC_rep1 (this study); NK H3K27ac: GSM4314409[4]; NK RNA-seq: MAP8.B6.Ly49m_WT_M6_RNA_rep1 (this study); CD8 Treg ATAC: CD8Treg_ATAC_rep1 (this study); CD8 Treg H3K27ac: GSM1876376[9]; CD8 Treg RNA-seq: GSM3758133[10].

b. Chromatin accessibility of the *MAP1s* of inhibitory *Ly49* genes and the *MAP8s* of activating *Ly49* genes in NK cells and CD8 Tregs, assayed by ATAC-seq. ATAC signals were normalized to the same sum across all MAPs (Methods). n = 3 (CD8 Treg) or 4 (NK) biologically independent samples. Means (bars) and individual values (points) are shown. Error bars: mean ± s.d. *P* values were FDR-adjusted. Two-tailed unpaired Student's *t*-test.

c. The expression of the inhibitory *B6.Ly49a* and *f*, and the activating *B6.Ly49h* and *m* in CD8 Tregs, assayed by RNA-seq. RNA-seq signals were normalized to the same sum across all *Ly49* genes (Methods). 3 CD8 Tregs samples were used. Data source: GSE130975[10].

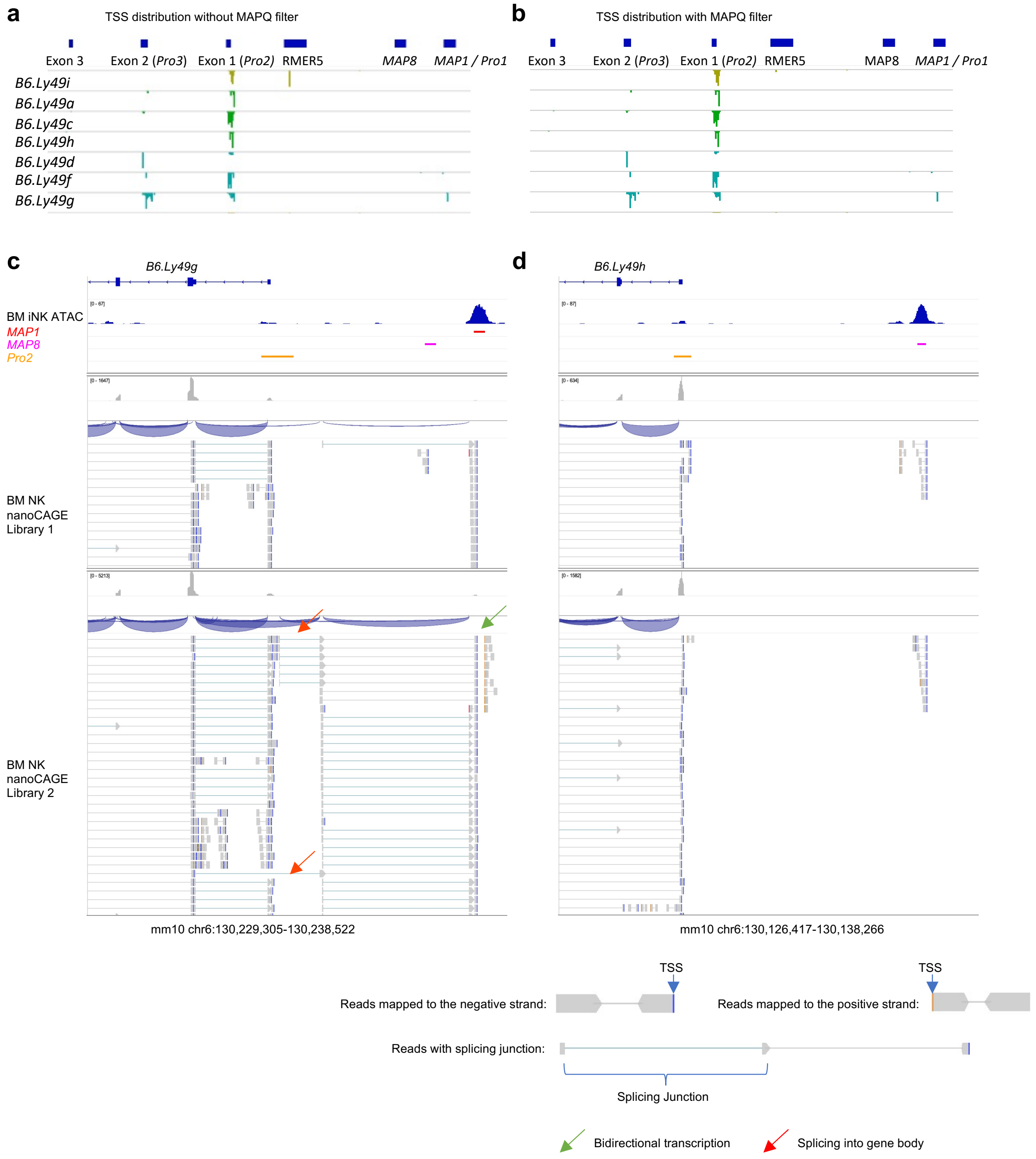

Supplementary Fig. 13 | *Ly49* TSS profiles in bone marrow NK cells.

a, Distribution of *Ly49* TSS signals along the *Ly49* consensus sequence. No MAPQ filter was applied. Library 2 was used (sorting strategy in Supplementary Fig. 7c).

b, Same as **a**, except that only reads passing MAPQ = 255 were used.

c-d, Raw reads view of *B6.Ly49g* and *B6.Ly49h* TSS. All reads shown passed the MAPQ = 255 filter. BM iNK ATAC: GSM2056300[7]. Source data are provided as a Source Data file.

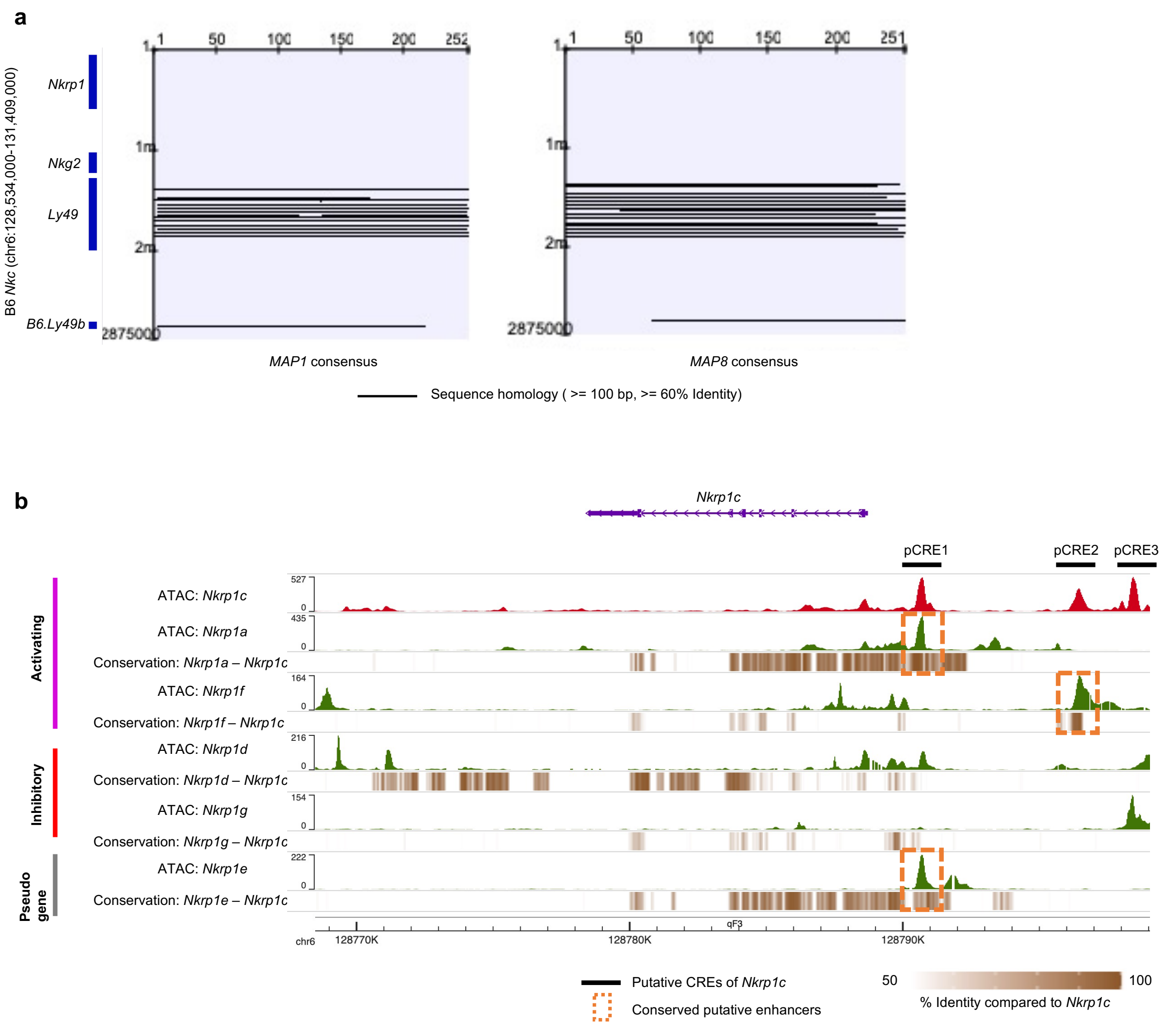

Supplementary Fig. 14 | *Cis*-regulatory evolution of the *Nkrp1* gene family.

a, Dot plots between the *Nkc* and the *MAP1* or *MAP8* consensus sequence (generated by UGENE). Homologous regions (black lines) were defined as regions >= 100 bp with >= 60% sequence identity.

b, Sequence conservation and ATAC-seq signals of *Nkrp1* genes (gene body ± 10 Kb), projected onto the *Nkrp1c* locus based on pairwise alignment (see Methods).

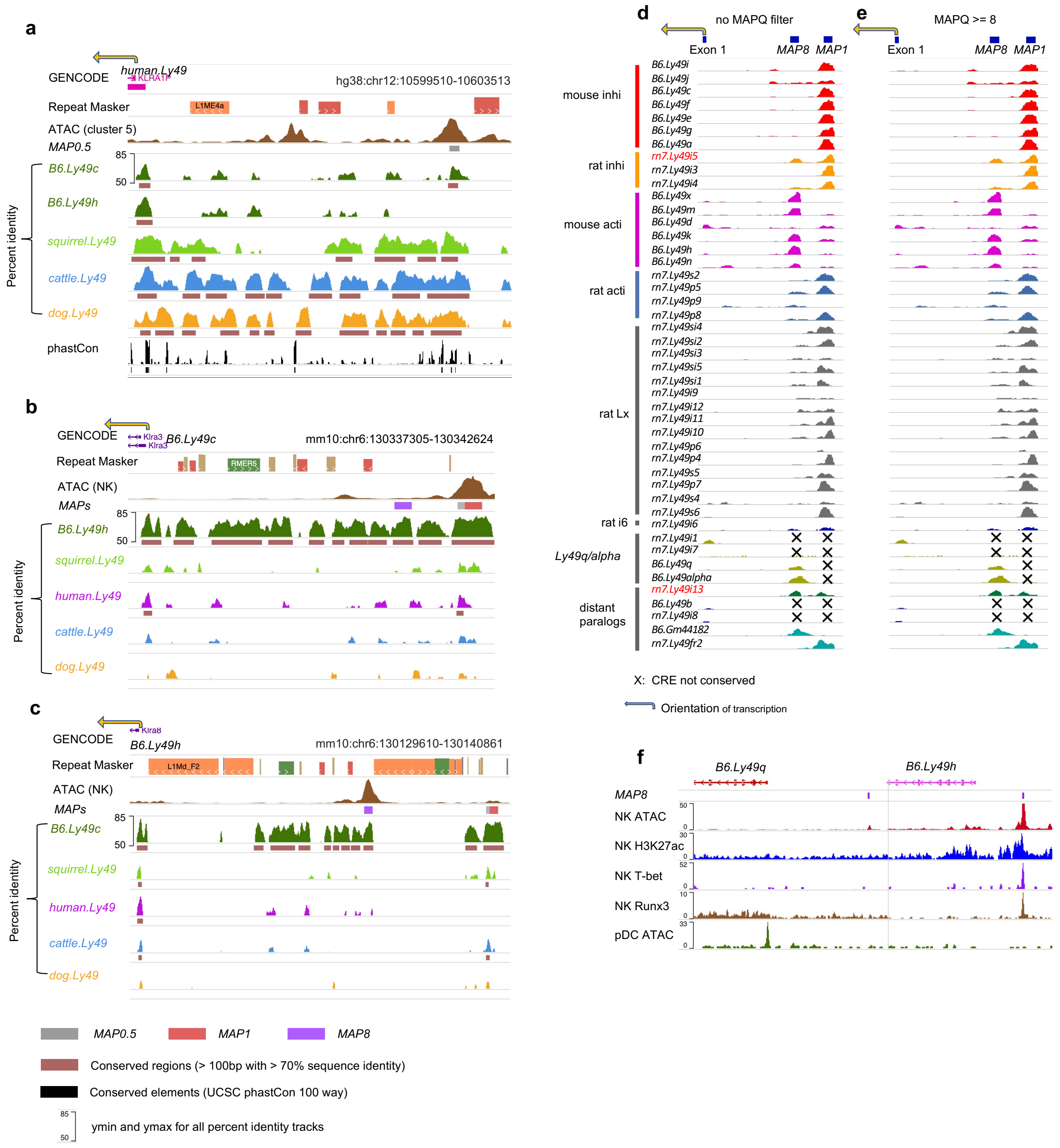

Supplementary Fig. 15 | Sequence conservation and chromatin accessibility of the *cis*-regulatory region upstream of *Ly49* genes.

a-c, Upstream region sequence conservation among *Ly49* genes in mice, squirrels, humans, cattle, and dogs. *Human.Ly49* (**a**), *B6.Ly49c* (**b**), and *B6.Ly49h* (**c**) were used as the reference to visualize percent sequence identity among *Ly49* genes, which was calculated by VISTA (Methods).

d-e, Similar to Fig. 2a and Supplementary Fig. 9a, but including rat NK cell ATAC-seq data. Only reads with 100% match to the references were used, without (**d**) or with (**e**) requiring MAPQ >= 8. *rn7.Ly49p1*, *p2*, *p3*, and *fr1* were excluded due to the absence of sequence at the plotted region.

rn7.Ly49i2 was excluded due to an inversion in the region. *rn7.Ly49s7* and *s8* were excluded because the closely related *rn7.Ly49s1*[11] is missing from the rn7 assembly, which could lead to incorrect alignment of reads from *s1* to *s7* or *s8*. Lx indicates the rat *Ly49* clade characterized by the presence of an Lx transposon in intron 2.

f, The ATAC-seq and H3K27ac landscapes of *B6.Ly49q*, compared to *B6.Ly49h*, in NK cells and pDCs, suggesting that *MAP8.B6.Ly49q* is likely non-functional, and that *B6.Ly49q* expression in pDCs[12] is likely driven by its core promoter. Data source: NK ATAC: the B6_Ly49Dp_ATAC_rep1 (Supplementary Data) sample from this study; NK H3K27ac: GSM4314409[4]; NK T-bet: GSM4314405[4]; NK Runx3: GSM1214531[6]; pDC ATAC: Immgen[13] (GSM2692341).

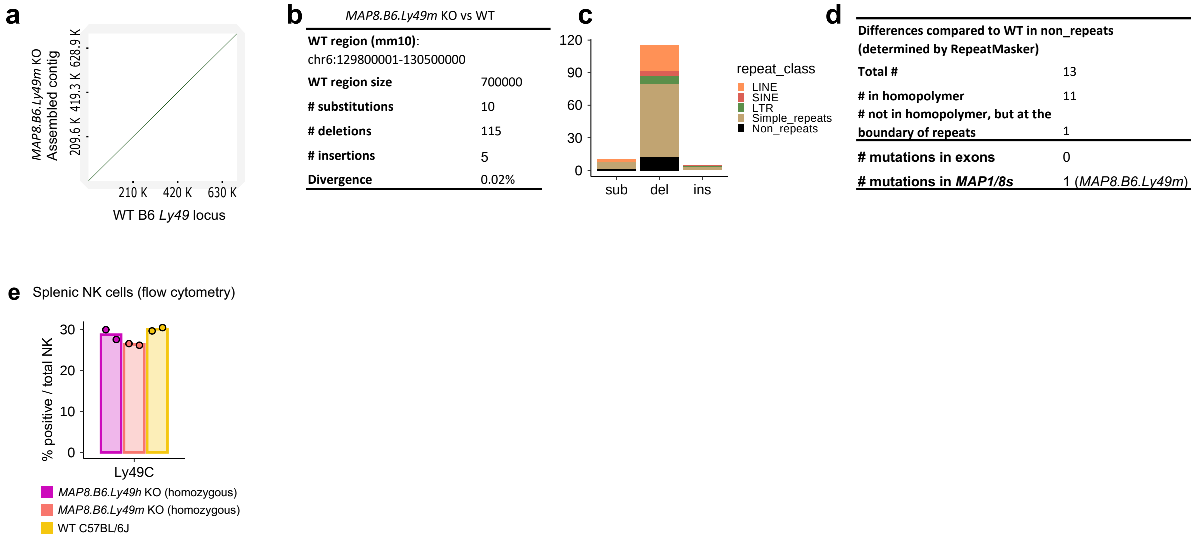

Supplementary Fig. 16 | *Ly49* locus assembly and Ly49C expression of *MAP8.B6.Ly49m* KO mice.

a, Same as Fig. 2g, but for the assembled *Ly49* locus of the *MAP8.B6.Ly49m* KO strain.

b-d, Same as Supplementary Fig. 9f-i, but for the assembled *Ly49* locus of the *MAP8.B6.Ly49m* KO strain.

e, The surface expression (measured by flow cytometry) of Ly49C in WT and KO animals. Means (bars) and individual values (points) are shown. n = 2 female animals. Source data are provided as a Source Data file.

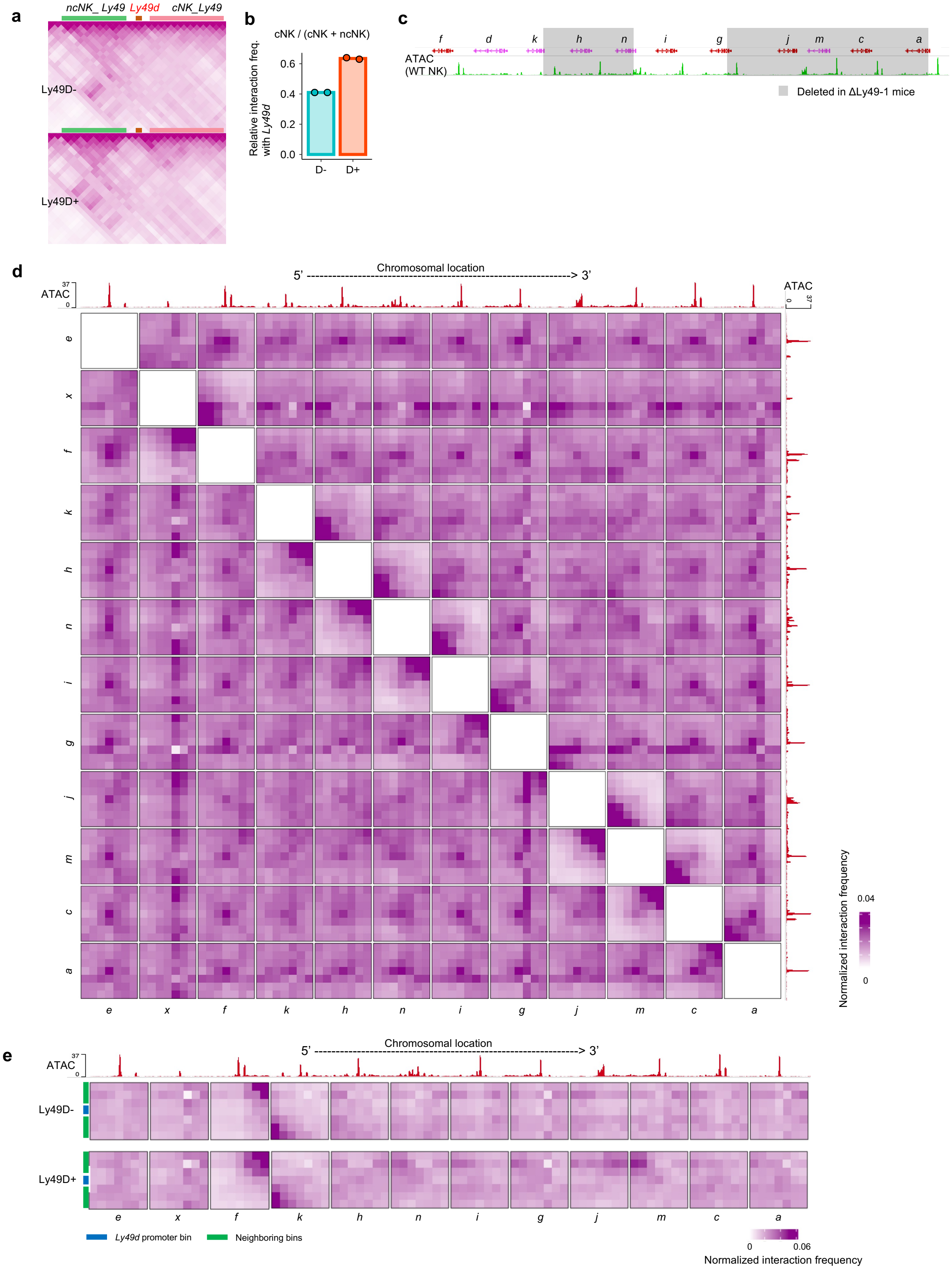

Supplementary Fig. 17 | MAP - MAP interactions and promoter - MAP interactions.

a, Large-scale interaction profiles of *B6.Ly49d* in Ly49D$^+$ vs Ly49D$^-$ cells, at 25 Kb resolution. *ncNK_Ly49* region (not expressed in conventional NK cells): mm10 chr6:129764477-130015298; *cNK_Ly49* region (expressed in conventional NK cells): mm10 chr6:130105696-130391935. See Methods.

b, Quantification of **a**. Means (bars) and individual values (points) are shown. n = 2 biologically independent samples.

c, The 2 regions deleted in ΔLy49-1 animals (shaded in grey). Both regions were deleted together in the same animal[14].

d, The interaction frequencies between each combination of 2 MAPs (and their neighboring bins) were extracted from the VC-normalized global contact matrix as a submatrix. Each such submatrix was then normalized to the sum of 1. For each combination of 2 MAPs, 4 such submatrices were generated, corresponding to the 4 samples (2 Ly49D$^+$ and 2 Ly49D$^-$). They were averaged and presented as 1 heatmap. Red tracks at the top and right of the plot are ATAC-seq profiles from the B6_Ly49Dp_ATAC_rep1 (Supplementary Data) sample.

e, Similar to **d**, the interaction frequencies between the *B6.Ly49d* promoter (and its neighborhood) and each MAP (and its neighborhood) were extracted from the global VC-normalized chromatin contact matrix. Each extracted submatrix was then normalized to the sum of 1. For each MAP under each condition, 2 such submatrices were generated, corresponding to the 2 biological replicates. They were averaged and presented as 1 heatmap. At the top: ATAC-seq profiles from the B6_Ly49Dp_ATAC_rep1 (Supplementary Data) sample.

Supplementary References

1. Wilhelm, B. T., Gagnier, L. & Mager, D. L. Sequence Analysis of the Ly49 Cluster in C57BL/6 Mice: A Rapidly Evolving Multigene Family in the Immune System. *Genomics* **80**, 646–661 (2002).

2. Makrigiannis, A. P., Patel, D., Goulet, M.-L., Dewar, K. & Anderson, S. K. Direct sequence comparison of two divergent class I MHC natural killer cell receptor haplotypes. *Genes Immun* **6**, 71–83 (2005).

3. Belanger, S., Tai, L.-H., Anderson, S. K. & Makrigiannis, A. P. Ly49 cluster sequence analysis in a mouse model of diabetes: an expanded repertoire of activating receptors in the NOD genome. *Genes Immun* **9**, 509–521 (2008).

4. Sciumè, G. *et al.* Rapid Enhancer Remodeling and Transcription Factor Repurposing Enable High Magnitude Gene Induction upon Acute Activation of NK Cells. *Immunity* (2020) doi:10.1016/j.immuni.2020.09.008.

5. Kissiov, D. U. *et al.* Binary outcomes of enhancer activity underlie stable random monoallelic expression. *Elife* **11**, e74204 (2022).

6. Lotem, J. *et al.* Runx3-mediated transcriptional program in cytotoxic lymphocytes. *Plos One* **8**, e80467 (2013).

7. Shih, H.-Y. *et al.* Developmental Acquisition of Regulomes Underlies Innate Lymphoid Cell Functionality. *Cell* **165**, 1120–1133 (2016).

8. Grant, C. E., Bailey, T. L. & Noble, W. S. FIMO: scanning for occurrences of a given motif. *Bioinformatics* **27**, 1017–1018 (2011).

9. Kim, H.-J. *et al.* Stable inhibitory activity of regulatory T cells requires the transcription factor Helios. *Science* **350**, 334–339 (2015).

10. Saligrama, N. *et al.* Opposing T cell responses in experimental autoimmune encephalomyelitis. *Nature* **572**, 481–487 (2019).

11. Nylenna, Ø. *et al.* The genes and gene organization of the Ly49 region of the rat natural killer cell gene complex. *Eur J Immunol* **35**, 261–272 (2005).

12. Kamogawa-Schifter, Y. *et al.* Ly49Q defines 2 pDC subsets in mice. *Blood* **105**, 2787–2792 (2005).

13. Yoshida, H. *et al.* The cis-Regulatory Atlas of the Mouse Immune System. *Cell* **176**, 897-912.e20 (2019).

14. Parikh, B. A. *et al.* Control of Viral Infection by Natural Killer Cell Inhibitory Receptors. *Cell Reports* **32**, 107969 (2020).
