# Supplementary material for: Cis-regulatory evolution of the recently expanded Ly49 gene family
Source: Nat Commun. 2024 Jun 6;15:4839. doi: 10.1038/s41467-024-48990-y (PMC11156856; doi:10.1038/s41467-024-48990-y)
Supplement: Supplementary file 4 — Reporting Summary [file 41467_2024_48990_MOESM4_ESM.pdf]

nature portfolio

Corresponding author(s): Sytse J. Piersma, Wayne M. Yokoyama, Ting Wang

Last updated by author(s): Apr 22, 2024

Reporting Summary

Nature Portfolio wishes to improve the reproducibility of the work that we publish. This form provides structure for consistency and transparency in reporting. For further information on Nature Portfolio policies, see our Editorial Policies and the Editorial Policy Checklist.

Statistics

For all statistical analyses, confirm that the following items are present in the figure legend, table legend, main text, or Methods section.

n/a	Confirmed	
☐	☒	The exact sample size (n) for each experimental group/condition, given as a discrete number and unit of measurement
☐	☒	A statement on whether measurements were taken from distinct samples or whether the same sample was measured repeatedly
☐	☒	The statistical test(s) used AND whether they are one- or two-sided<br>*Only common tests should be described solely by name; describe more complex techniques in the Methods section.*
☒	☐	A description of all covariates tested
☐	☒	A description of any assumptions or corrections, such as tests of normality and adjustment for multiple comparisons
☐	☒	A full description of the statistical parameters including central tendency (e.g. means) or other basic estimates (e.g. regression coefficient) AND variation (e.g. standard deviation) or associated estimates of uncertainty (e.g. confidence intervals)
☐	☒	For null hypothesis testing, the test statistic (e.g. F, t, r) with confidence intervals, effect sizes, degrees of freedom and P value noted<br>*Give P values as exact values whenever suitable.*
☐	☒	For Bayesian analysis, information on the choice of priors and Markov chain Monte Carlo settings
☒	☐	For hierarchical and complex designs, identification of the appropriate level for tests and full reporting of outcomes
☐	☒	Estimates of effect sizes (e.g. Cohen's d, Pearson's r), indicating how they were calculated

Our web collection on statistics for biologists contains articles on many of the points above.

Software and code

Policy information about availability of computer code

Data collection	BD FACSDiva (v8.0.1); Cytek SpectroFlo (v3.1)
Data analysis	All custom code is collected at: https://github.com/ChangxuFan/Ly49evolution<br><br>command line: R (v3.6.1); AIAP (v1.1); TagDust2 (v2.33); Cutadapt (v1.18); STAR (v2.5.4b); UMI-tools (v1.0.1); samtools (v1.7); sambamba (v0.7.1); methylQA (v0.2.1); Bowtie2 (v2.3.4.1); FastQC (v0.11.9); fastp (v0.20.0); deepTools (v3.5.0); macs2 (v2.1.1.20160309); subread (v2.0.0); cellranger-arc (v2.0); juicer (v1.6); MAFFT (v7.427); VISTA (v1.4.26); MrBayes (v3.2); cellranger (v6.0); Guppy (v6.4.6); minimap2 (v2.24); flye (v2.9.2).<br><br>R packages: CAGEr (v1.30.3); DESeq2 (v1.26.0); edgeR (v3.26.8); Seurat (v3.2.3); ArchR (v1.0.1); Biostrings (v2.54.0); ggplot2 (v3.3.3); GenomicRanges (v1.38.0); GenomicAlignments (v1.22.1); GenomicInteractions (v1.20.3); SummarizedExperiment (v1.16.1); ComplexHeatmap (v2.8.0); InteractionSet (v1.14.0); rtracklayer (v1.46.0).<br><br>webtools: WashU Epigenome Browser (v54.0.6); agilent suredesign (https://earray.chem.agilent.com/suredesign/); D-Genies (v1.5.0)<br><br>Desktop software: IGV (v2.13.2); FlowJo (v10.4)

For manuscripts utilizing custom algorithms or software that are central to the research but not yet described in published literature, software must be made available to editors and reviewers. We strongly encourage code deposition in a community repository (e.g. GitHub). See the Nature Portfolio guidelines for submitting code & software for further information.

Data

Policy information about availability of data

All manuscripts must include a data availability statement. This statement should provide the following information, where applicable:

- Accession codes, unique identifiers, or web links for publicly available datasets
- A description of any restrictions on data availability
- For clinical datasets or third party data, please ensure that the statement adheres to our policy

All sequencing data generated in this study have been deposited in GEO DataSets under the accession code GSE226502 [https://www.ncbi.nlm.nih.gov/geo/query/acc.cgi?acc=GSE226502], and are publicly available. Other datasets used in this study are also publicly available at GEO DataSets:
Mouse CD8 Treg:
RNA-seq: GSE133364 [https://www.ncbi.nlm.nih.gov/geo/query/acc.cgi?acc=GSE133364]; H3K27ac: GSM1876376 [https://www.ncbi.nlm.nih.gov/geo/query/acc.cgi?acc=GSM1876376].

Mouse NK ChIP-seq:
H3K4me3: GSM4314407 [https://www.ncbi.nlm.nih.gov/geo/query/acc.cgi?acc=GSM4314407] and GSM4314396 [https://www.ncbi.nlm.nih.gov/geo/query/acc.cgi?acc=GSM4314396]; H3K27ac: GSM4314409 [https://www.ncbi.nlm.nih.gov/geo/query/acc.cgi?acc=GSM4314409]; p300: GSM2056372 [https://www.ncbi.nlm.nih.gov/geo/query/acc.cgi?acc=GSM2056372]; T-bet: GSM4314405 [https://www.ncbi.nlm.nih.gov/geo/query/acc.cgi?acc=GSM4314405]; Runx3: GSM1214531 [https://www.ncbi.nlm.nih.gov/geo/query/acc.cgi?acc=GSM1214531].
Human T-bet ChIP-seq: GSM776557 [https://www.ncbi.nlm.nih.gov/geo/query/acc.cgi?acc=GSM776557].

Cattle: spleen ATAC: GSM4799634 [https://www.ncbi.nlm.nih.gov/geo/query/acc.cgi?acc=GSM4799634].
Dog spleen ATAC: SRX5812510 [https://www.ncbi.nlm.nih.gov/sra/?term=SRX5812510].
Cattle RNA-seq: GSE158430 [https://www.ncbi.nlm.nih.gov/geo/query/acc.cgi?acc=GSE158430].
Dog RNA-seq: barkbase [https://data.broadinstitute.org/barkbase/RNA-seq/].
Golden hamster genome annotation: GSE200596 [https://www.ncbi.nlm.nih.gov/geo/query/acc.cgi?acc= GSE200596]
ATAC-seq from NKG2A+ NK cells of the F1 progeny of the B6 x BALB/c cross: GSM5492291 [https://www.ncbi.nlm.nih.gov/geo/query/acc.cgi?acc=GSM5492291].
Mouse iNK ATAC: GSM2056300 [https://www.ncbi.nlm.nih.gov/geo/query/acc.cgi?acc=GSM2056300].
Mouse pDC ATAC: GSM2692341 [https://www.ncbi.nlm.nih.gov/geo/query/acc.cgi?acc=GSM2692341].
Mouse splenic NK single-cell RNA-seq: GSE189807 [https://www.ncbi.nlm.nih.gov/geo/query/acc.cgi?acc= GSE189807]

Human peripheral blood single cell multiome data is publicly available from 10x website [https://www.10xgenomics.com/datasets/pbmc-from-a-healthy-donor-granulocytes-removed-through-cell-sorting-10-k-1-standard-2-0-0]. Source data are provided with this paper. Flow cytometry data are available in the Source Data file.

Research involving human participants, their data, or biological material

Policy information about studies with human participants or human data. See also policy information about sex, gender (identity/presentation), and sexual orientation and race, ethnicity and racism.

Reporting on sex and gender	no human research
Reporting on race, ethnicity, or other socially relevant groupings	no human research
Population characteristics	no human research
Recruitment	no human research
Ethics oversight	no human research

Note that full information on the approval of the study protocol must also be provided in the manuscript.

Field-specific reporting

Please select the one below that is the best fit for your research. If you are not sure, read the appropriate sections before making your selection.

☒ Life sciences ☐ Behavioural & social sciences ☐ Ecological, evolutionary & environmental sciences

For a reference copy of the document with all sections, see nature.com/documents/nr-reporting-summary-flat.pdf

Life sciences study design

All studies must disclose on these points even when the disclosure is negative.

Sample size	No statistical methods were used to predetermine sample sizes. Sample sizes were chosen according to field standards (as seen in previous

literature) established for the same assays. Specifically:

MCMV infection was performed using 11 KO and 15 WT mice, divided into 2 separate experiments, to account for the variation in viral titer measurements, as established in previous publications (PMID: 32723479; 26720279).

Key flow cytometry analyses were performed using at least 3 animals in each group, as established by previous studies using flow cytometry to measure Ly49 expression (PMID: 35617021).

Next-gen sequencing based studies were performed using 2-3 biologically independent samples, as established previously (PMID: 25516281), with the exception of nanoCAGE and WGBS experiments for splenic NK cells. These nanoCAGE/WGBS experiments were performed only once to confirm previously reported findings.

Data exclusions

The following genes were excluded from the Ly49 gene tree due to the absence or incompleteness of sequences at intron 1 and intron 2: 129.Ly49alpha, 129.Ly49ui, 129.Ly49u, 129.Ly49q3, NOD.Ly49x, NOD.Ly49alpha2, rn7.Ly49p1, rn7.Ly49p2, rn7.Ly49p3, rn7.Ly49fr1.

Replication

For both KO mouse models, phenotypes were confirmed in at least 3 founders.

Randomization

No randomization was performed. Randomization was not relevant to the study because samples were grouped based on genotype, which cannot be randomized.

Blinding

Not relevant: no subjective measurements.

Reporting for specific materials, systems and methods

We require information from authors about some types of materials, experimental systems and methods used in many studies. Here, indicate whether each material, system or method listed is relevant to your study. If you are not sure if a list item applies to your research, read the appropriate section before selecting a response.

Materials & experimental systems

n/a	Involved in the study	
☐	☒	Antibodies
☐	☒	Eukaryotic cell lines
☒	☐	Palaeontology and archaeology
☐	☒	Animals and other organisms
☒	☐	Clinical data
☒	☐	Dual use research of concern
☒	☐	Plants

Methods

n/a	Involved in the study	
☒	☐	ChIP-seq
☐	☒	Flow cytometry
☒	☐	MRI-based neuroimaging

Antibodies

Antibodies used

All antibodies were used with 1:100 dilution, unless otherwise noted.
Antibodies for NK cells: The following antibodies and markers were used (anti-mouse unless otherwise indicated):
From BD Biosciences: CD49a (Ha31/8; PE; Cat# 562115), Ly49A (A1; Biotin; Cat# 557423), Ly49F (HBF-719; PE; Cat# 550987), NK1.1 (PK136, PE-Cy7; Cat# 552878), Streptavidin (PE; Cat# 554061);
From BioLegend: Ly49A (YE1/48.10.6; FITC; Cat# 116805), Ly49H (3D10; AF647; Cat# 144710), NK1.1 (PK136; BV650; Cat# 108735), Rat CD3 (1F4; PerCP-Cy5.5; Cat# 201417), Rat NKp46 (CD335) (WEN23; PE; Cat# 250803), Rat NKR-P1 (CD161) (3.2.3; APC; Cat# 205606), CD11b (M1/70, BV421; Cat# 101236; 1:200 dilution), Ly49C (4LO3311; AF647; custom conjugation via BioLegend; 1:50 dilution);
From Thermo Fisher: CD11b (M1/70; eF450; Cat# 48-0112-82), CD27 (LG.7F9; PE-Cy7, APC; Cat# 25-0271-82, 17-0271-82), CD3 (145-2C11; APC-eF780; Cat# 47-0031-82), CD19 (eBio1D3; APC-eF780; Cat# 47-0193-82), CD4 (RM4-5; APC-eF780; Cat# 47-0042-82), CD49b (DX5; eF450; Cat# 48-5971-82), CD8 (53-6.7; APC-eF780; Cat# 47-0081-82), CD94 (18d3; eF450; Cat# 48-0941-82), Ly49D (4D11; APC; Cat# 17-5782-82), Ly49E/F (CM4; PerCP; 46-5848-82), Ly49G2 (eBio4D11; FITC; Cat# 11-5781-82), Ly49H (3D10; FITC; Cat# 11-5886-82), Ly49I (YLI-90, FITC; Cat# 11-5895-85) NKG2A/C/E (20D5; FITC; Cat# 11-5896-85), NKG2AB6 (16a11; PerCP-eF710; Cat# 46-5897-82), NKp46 (29A1.4; PE-Cy7 1:25 dilution, PerCP-eF710, PE-eF610; Cat# 25-3351-82, 46-3351-82, 61-3351-82), TCRB (H597; APC-eF780; 47-5961-82), CD122 (TM-b1; PE; Cat# 12-1222-82), Viability (eF506; 65-0866; 1:500 dilution), Rat CD3 (eBioG4.18 (G4.18); Biotin; Cat# 13-0030-82; 1:250 dilution), Rat CD45R (B220) (HIS24; Biotin; Cat# 13-0460-82; 1:250 dilution), Ly49I (YLI-90; Biotin; Cat# MA5-28667);
From Jackson ImmunoResearch: anti-IgG3 (AF647; Cat# 115-605-209);
From Leinco: Ly49C (4LO3311, PE, Cat# L312).
Antibodies for CD8 Tregs: The following biotinylated antibodies were used for negative selection of CD8 Tregs (all from BioLegend): CD4 (clone RM4-5; Cat# 100508), CD19 (clone MB19-1; Cat# 101504), Ly-6G (clone 1A8; Cat# 127604), F4/80 (clone BM8; Cat# 123106), and CD14 (clone Sa14-2; Cat# 123306).
The following antibodies and markers were used for sorting CD8 Tregs:
From BioLegend: CD4 (RM4-5; Alexa Fluor 700; Cat# 100536), CD8a (53-6.7; APC-Cy7; Cat# 100714), CD44 (IM7; PerCP; Cat# 103036; 1:50 dilution), Ly49C/F/I/H (14B11; PE; Cat# 108208), Ly49H (3D10; PE; Cat# 144706), Ly49D (4E5; PE; Cat# 138308), CD19 (6D5; PE-Cy7; Cat# 115520), CD3 (17A2; APC; Cat# 100236; 1:50 dilution);

	From BD Biosciences: CD122 (TM-β1; BV421; Cat# 752988), Ly49F (HBF-719; PE; Cat# 550987), Ly49A (A1; PE; Cat# 557424);<br>From Invitrogen: Ly49I (YLI-90; PE; Cat# 12-5895-82); LIVE/DEAD Aqua Dead Cell Stain Kit (Cat# L34957);<br>From Miltenyi: Ly49G2 (REA1053; PE; Cat# 130-118-033), Ly49C/I (REAL296; PE; Cat# 130-118-940), Ly49E/F (REAL331; PE; Cat# 130-118-530; 1:50 dilution).
Validation	Antibodies used in this study have been previously characterized and were obtained from commercial vendors. According to the vendors, rigorous QC for each lot is performed. Specific statements of validation include:<br><br>BD Biosciences:<br>“The specificity is confirmed using multiple methodologies that may include a combination of flow cytometry, immunofluorescence, immunohistochemistry or western blot to test staining on a combination of primary cells, cell lines or transfectant models.<br>All flow cytometry reagents are titrated on the relevant positive or negative cells. To save time and cell samples for researchers, test size reagents are bottled at an optimal concentration with the best signal-to-noise ratio on relevant models during the product development. To ensure consistent performance from lot-to-lot, each reagent is bottled to match the previous lot MFI.<br>Technical data sheets provide data generated on the relevant primary model at this optimal concentration based on a titration curve. QC data on any lot of reagent can be requested through ResearchApplications@bd.com.”<br>[https://www.bdbiosciences.com/en-us/products/reagents/flow-cytometry-reagents/research-reagents/quality-and-reproducibility]<br><br>BioLegend:<br>“Specificity testing of 1-3 target cell types with either single- or multi-color analysis (including positive and negative cell types). Once specificity is confirmed, each new lot must perform with similar intensity to the in-date reference lot. Brightness (MFI) is evaluated from both positive and negative populations. Each lot product is validated by QC testing with a series of titration dilutions.”<br>[https://www.biolegend.com/en-us/quality/quality-control]<br><br>Thermo Fisher:<br>"The IWGAV has proposed five approaches for antibody validation: using genetics; using an orthogonal (non-antibody) strategy; using independent antibodies binding to the same target; correlating antibody labelling with the expression of tagged proteins; and immunoprecipitation followed by mass spectrometry. At least one of these strategies should be used when validating an antibody for a specific application. Thermo Fisher has used these recommendations as the basis for its own internal systematic approach for verifying the specificity and functionality of antibodies. Detailed testing protocols and results, as well as published antibody data, are collated on the company’s website."<br>[https://assets.thermofisher.com/TFS-Assets/BID/Reference-Materials/antibody-reproducibility-validation-specificity-tests-nature-articles.pdf]<br><br>Leinco Ly49C (Cat# L312):<br>PMID: 32726632<br><br>Jackson anti-IgG3 (Cat# 115-605-209):<br>PMID: 34282664<br><br>Miltenyi:<br>“All antibodies are tested for lot-to-lot consistency at two stages, during the antibody raw material production as well as the fluorochrome-conjugation process. During development of an antibody, a suitable test to verify specificity of the clone is performed. All conjugated antibodies, including multiple conjugates of the same clone, are tested on primary samples. Whenever possible, antibodies are tested in multicolor panels. In addition, antibodies are routinely tested on cells derived from tissues using enzymatic treatment.”<br>[https://www.miltenyibiotec.com/US-en/products/macs-antibodies/antibody-validation.html]

Eukaryotic cell lines

Policy information about cell lines and Sex and Gender in Research

Cell line source(s)	EL4 (ATCC TIB-39): EL4 is a T lymphoblast that was established from a lymphoma induced in a C57BL mouse by 9,10-dimethyl-1,2-benzanthracene.
Authentication	The EL4 cell line was not authenticated (not relevant to study)
Mycoplasma contamination	Not tested for Mycoplasma contamination
Commonly misidentified lines (See ICLAC register)	No commonly misidentified lines used in this study. The EL4 cell line used is not within the ICLAC register.

Animals and other research organisms

Policy information about studies involving animals; ARRIVE guidelines recommended for reporting animal research, and Sex and Gender in Research

Laboratory animals	Mus musculus: (C57BL/6J; 129S6/SvEvTac; NOD/ShiLTJ; CB6F1/J). Rattus norvegicus (BN/NHsdMcwi).

Mouse models generated in this study: MAP8.B6.Ly49h KO, MAP8.B6.Ly49m KO (both on the B6 background).
All animals used are between 8-16 weeks old. The age for each individual animal is detailed in Source Data

Wild animals

This study did not involve wild animals

Reporting on sex

Animal sex was determined by vendors, or determined according to the appearance of nipples and genital spacing.
The phenotypes of KO animals generated in this study have been confirmed in both male and female mice. No sex-based analysis was performed, because sex-specific phenotypes in the regulation Ly49 expression was not seen in this study or previous work (PMID: 35617021; 14707081; 15345220).
The sex for individual animals can be found in the Source Data.

Field-collected samples

This study did not involve samples collected from the field.

Ethics oversight

The studies were approved by the Animal Studies Committee at Washington University School of Medicine under animal protocol 21-0090.

Note that full information on the approval of the study protocol must also be provided in the manuscript.

Plants

Seed stocks

No plants involved.

Novel plant genotypes

No plants involved.

Authentication

No plants involved.

Flow Cytometry

Plots

Confirm that:

- [x] The axis labels state the marker and fluorochrome used (e.g. CD4-FITC).
- [x] The axis scales are clearly visible. Include numbers along axes only for bottom left plot of group (a 'group' is an analysis of identical markers).
- [x] All plots are contour plots with outliers or pseudocolor plots.
- [x] A numerical value for number of cells or percentage (with statistics) is provided.

Methodology

Sample preparation

Mouse NK cells (peripheral blood and spleen): Cheek-blood was collected in 50mM EDTA-tubes, red blood cells were lysed with Tris-NH4Cl RBC lysis buffer and remaining cells were analyzed by flow cytometry. Single cell splenocyte solutions were obtained from spleens using cell strainers and treated with RBC lysis buffer. Where sorting was performed, NK cells were pre-enriched using the EasySep mouse NK cell isolation kit (Stemcell Technologies), followed by surface staining with indicated antibodies in 2.4G2 hybridoma supernatant to block Fc receptors. NK cells were sorted as singlet CD4-CD8-TCRβ-CD19-NK1.1+NKp46+CD49a-CD49b+ (B6, CB6F1/J, and KO animals generated in this study) or singlet CD4-CD8-TCRβ-CD19-NKp46+CD49a-CD49b+ (129 and NOD, which do not encode NK1.1 in their genomes). Cells were sorted on a FACSAria (BD Biosciences) into RPMI with 10% FBS for downstream library preparation. For phenotypic analysis without sorting, splenocyte or blood samples were stained with fixable viability dye (Thermo Fisher Scientific), continued by staining of cell surface molecules in 2.4G2 supernatant, followed by secondary antibodies and streptavidin. Where needed, samples were fixed in 1% paraformaldehyde until acquisition. Samples were acquired using FACSCanto (BD Biosciences) and analyzed using FlowJo (v10.4 BD Biosciences). NK cells were defined as singlet viability-CD4-CD8-TCRβ-CD19-NK1.1+NKp46+.

Mouse NK cells (bone marrow): To test the effect of MAP8 deletion on B6.Ly49h expression, cells were collected from mouse tibiae through cutting off the distal end of the tibiae and centrifuging the tibiae at 800 g for 3 minutes. RBC lysis and antibody staining were then performed similar to splenocytes. NK cells were identified as CD3-CD19-CD122+NK1.1+. NKp46, CD27, and CD11b were used to delineate NK cell maturation trajectory. To sort bone marrow NK cells for nanoCAGE, two strategies were tested: for library 1, NK cells were pre-enriched through negative selection similar to splenic NK cells, and sorted as CD3-CD122+NK1.1+CD49b(DX5)low. For library 2, NK cells were sorted as CD3-CD122+NK1.1+CD49b(DX5)high, without negative selection based enrichment.

Mouse NK cells (spleen, licensed vs unlicensed): NK cells were isolated as aforementioned for spleen, but identified using CD3-CD19-NK1.1+NKp46+. NKG2A, Ly49C, and Ly49I were used to identify licensed vs unlicensed NK cells.
Rat NK cells: Spleens were homogenized using cell strainers and treated with RBC lysis buffer to obtain single cell splenocyte

	solutions. To enrich for NK cells, splenocytes were stained with Biotinylated CD3 and CD45R (B220)-specific antibodies, followed by incubation with streptavidin RapidSpheres (Stemcell Technologies). CD3+ and CD45R+ cells were subsequently depleted using magnets. Pre-enriched NK cells were then stained with streptavidin-BV421 and anti-CD3, anti-NKp46, and anti-NKR-P1. Rat NK cells were defined as singlet CD3-CD45R-NKp46+NKRP1+ and sorted similar to mouse cells.<br><br>Mouse CD8 Tregs: Single cell splenocyte solutions were obtained from spleens using cell strainers and treated with RBC lysis buffer. Next, negative selection was performed using Akadeum microbubbles (cat# 11110-000) and biotinylated antibodies listed above. Following surface staining, CD8 Tregs were sorted as singlet live CD3+CD19-CD8a+CD44+CD122+Ly49+. Cells were sorted using FacsAriaII (BD Biosciences).
Instrument	FACSCanto (BD Biosciences); FACSAria (BD Biosciences); Cytek Aurora 5 Laser (Cytek)
Software	BD FACSDiva (v8.0.1); Cytek SpectroFlo (v3.1)
Cell population abundance	After sorting, sorted cells were re-run through flow cytometry to assess if sorted cells were from the desired gates. For most sorting experiments, >99% cells were from the desired gate.
Gating strategy	Lymphocytes were selected based on FSC-A/SSC-A; single cells were selected based on FSC-A/FSC-H and SSC-A/SSC-H.<br>For sorting, NK cells were defined as singlet CD4-CD8-TCRβ-CD19-NK1.1+NKp46+CD49a-CD49b+ (B6, CB6F1/J, and KO animals generated in this study), singlet CD4-CD8-TCRβ-CD19-NKp46+CD49a-CD49b+ (129 and NOD), or singlet CD3-CD45R-NKp46+NKRP1+ (rat). For flow cytometry staining without sorting, B6 NK cells were defined as singlet viability-CD4-CD8-TCRβ-CD19-NK1.1+NKp46+<br><br>Mouse NK cells (spleen, licensed vs unlicensed): NK cells were identified using CD3-CD19-NK1.1+NKp46+. NKG2A, Ly49C, and Ly49I were used to identify licensed vs unlicensed NK cells.<br><br>CD8 Tregs were sorted as singlet live CD3+CD19-CD8a+CD44+CD122+Ly49+.<br><br>To sort bone marrow NK cells for nanoCAGE, two strategies were tested: for library 1, NK cells were pre-enriched through negative selection similar to splenic NK cells, and sorted as CD3-CD122+NK1.1+CD49b(DX5)low. For library 2, NK cells were sorted as CD3-CD122+NK1.1+CD49b(DX5)high, without negative selection based enrichment.

☒ Tick this box to confirm that a figure exemplifying the gating strategy is provided in the Supplementary Information.
